# Marigold breeding in India: a comprehensive review of genetic advances, techniques and future prospects

**DOI:** 10.3389/fpls.2025.1619375

**Published:** 2025-09-26

**Authors:** Venkatesan D., Ramesh Kumar S.

**Affiliations:** Department of Horticulture and Food Science, VIT School of Agricultural Innovations and Advanced Learning (VAIAL), Vellore Institute of Technology, Vellore, Tamil Nadu, India

**Keywords:** breeding, pedigree selection, landscape, mutation breeding, tissue culture

## Abstract

Marigold (*Tagetes* spp.) is economically important flower crop widely cultivated for its vibrant flowers, use in religious ceremonies, landscaping, and extraction of carotenoids for industrial and pharmaceutical applications. Breeding advancements in marigold have primarily focused on enhancing yield, flower quality, and resistance to biotic and abiotic stresses. This review presents a comprehensive overview of progress in marigold breeding, covering traditional approaches such as selection, hybridization, and mutation breeding, as well as modern biotechnological tools, including marker-assisted selection (MAS), genomic selection, and CRISPR-based genome editing. Conventional breeding has led to the development of several high-yielding hybrids, including ‘Arka Abhi’ and ‘Arka Shubha,’ which are widely cultivated across India. Modern molecular approaches have facilitated the identification of quantitative trait loci (QTLs) associated with essential traits, improving the efficiency of breeding programs. In recent years, tissue culture techniques have played a pivotal role in the rapid propagation of elite varieties and the generation of somaclonal variants with desirable traits. Major challenges like a limited genetic base, climate change, pests and diseases still make sustainable production difficult. The integration of wild germplasm and advanced genomic tools offers promising avenues for addressing these limitations. Participatory breeding and interdisciplinary research play a crucial role in addressing location-specific demands and improving the economic viability of marigold cultivation. This analysis indicates the importance for sustainable breeding practices that match with growing market requirements and environmental issues. Hence, by integrating traditional knowledge with cutting-edge technologies, marigold breeding programs can unlock the crop’s full potential, contributing to the growth of India’s ornamental and agricultural sectors.

## 1 Introduction

In India, marigold (*Tagetes* spp.) is an attractive crop with significant cultural, economic, and industrial value. Valued for their vibrant flowers, marigolds hold a major role in religious and social ceremonies, as well as in landscaping. Marigold is native to South and Central America especially Mexico and belong to the family Asteraceae (Compositae) (Shafiee et al., 2023). The genus Tagetes commonly cultivated species are *Tagetes erecta* (African Marigold), *Tagetes patula* (French Marigold) and *Tagetes minuta*. Marigold are long upright and quick growing habit (Udchachon et al., 2021). The height of plants ranges from 30 to 90 cm (Yadav and Dahiya, 2020). The flowers of these varieties are deep orange, light orange, golden yellow, bright yellow and lemon yellow in color. The size of flower may vary from 4 to 6cm in diameter. Marigold is good source of carotenoid pigment for poultry feed to intensify yellow color of egg yolks (Zhang et al., 2020). The crop is a critical raw material for the extraction of carotenoids, particularly lutein and zeaxanthin, which are widely used in the dye, food, and pharmaceutical industries (Shafique et al., 2021). India is the leading producers of marigold, with major cultivation areas in Karnataka, Tamil Nadu, and Maharashtra contributing significantly to domestic and export markets. Marigold cultivation is successful because it can adapt to a variety of agroclimatic conditions and yield large quantities of flowers quickly (Kumar et al., 2020). The crop is grown both as a field crop and in protected environments, supporting livelihoods across various regions. Improved varieties and hybrids such as ‘Arka Bangara,’ ‘Arka Agni,’ and ‘Pusa Narangi Gainda’ have enhanced marigold production, meeting the growing demands for high-quality flowers (Kaushal et al., 2024). Marigold also finds industrial application like preparation of natural dyes and essential oils. It is used as mosquito and nematode repellents. The marigold plants are highly useful for suppressing the population of nematodes in the field. The uses of marigold are extensive, often referred to as, “Versatile crop with golden harvest”. Marigolds produce thiopenes, which are toxic to nematodes and used as trap crop in tomato, brinjal, tobacco etc. Despite its adaptability, marigold cultivation faces challenges such as the limited availability of high-performing hybrids and susceptibility to pests like aphids and diseases such as alternaria blight and powdery mildew (Dedhia, 2024). Climate-induced stress factors, including drought and salinity, significantly impact flower yield and quality, further emphasizing the need for stress-resilient varieties (Akhtar et al., 2022). Efforts to enhance marigold cultivation have shifted from traditional breeding approaches to incorporating molecular tools, including marker-assisted selection (MAS) and genome editing, to develop superior varieties. This integration of traditional and modern techniques is essential to overcoming current challenges and unlocking the crop’s full potential for sustainable production (Susrama and Yuliadhi, 2020). The state-wise Marigold production in India for 2022–23 is presented in **Table 1**.

**Table 1:** State-wise marigold production in India (2022-23).

State	Area (ha)	Production (MT)	Major varieties
Karnataka	15,000	120,000	Arka Bangara, Arka Basant
Tamil Nadu	12,000	90,000	CO-1, CO-2
West Bengal	10,000	80,000	Local cultivars
Maharashtra	8,500	60,000	African hybrids
Andhra Pradesh	7,000	55,000	Pusa Narangi

Source: (Source: NHB, 2022)

## 2 Methodology

A comprehensive review of the literature was carried out to collect scientific studies on the taxonomy, breeding objectives, conventional and modern methods of breeding in marigold. Research and review articles published up to December 2024 were sourced from databases like Scopus, Google Scholar, ScienceDirect, and others. Keywords like marigold breeding, pedigree selection, mutation breeding and micropropagation of marigold were used to guide the search. Studies that were out of scope, lacked sufficient details, or did not meet the relevance criteria were excluded. The selection process focused on relevance and relatedness to ensure only the most significant and appropriate information was included.

## 3 Taxonomy and genetic diversity

The genus *Tagetes*, belonging to the family Asteraceae, comprises approximately 50 species, with *Tagetes erecta* (African marigold) and *Tagetes patula* (French marigold) being the most widely cultivated species in India. These species differ in their morphology, flowering characteristics, and adaptability. *Tagetes erecta* is characterized by large flowers, upright growth habit, and suitability for cut flower production, while *Tagetes patula* produces small flowers and is favored for ornamental bedding purposes (Poulose et al., 2020). Taxonomic classification of *Tagetes* has traditionally relied on morphological traits, such as plant height, flower size, and leaf structure (Kaushal et al., 2024). However, recent advancements in molecular tools have provided a deeper understanding of genetic relationships within the genus. Highlighting the genetic proximity between species through phylogenetic analysis with molecular markers such as Random Amplified Polymorphic DNA (RAPD) and Simple Sequence Repeats (SSRs) enables breeders to take advantage of interspecific hybridization for transferring desired traits (Pangaribuan et al., 2022). Comparative genomic studies have also revealed that species within *Tagetes* share conserved genomic regions associated with traits like flower color and carotenoid biosynthesis, further aiding targeted breeding efforts (Cai et al., 2024).

Genetic diversity is a critical factor for the success of breeding programs, as it determines the extent of variation available for selection and hybridization. Assessments of genetic variability in marigold have been conducted using morphological, biochemical, and molecular markers (Namita et al., 2013). High levels of genetic diversity have been reported among marigold genotypes cultivated in different agro-climatic zones of India, reflecting the adaptability and evolutionary history. Molecular marker studies have been particularly effective in quantifying genetic diversity (Panwar et al., 2017). Efforts to conserve genetic resources of marigold are also gaining importance. Germplasm collections maintained by research institutes, such as the Indian Council of Agricultural Research (ICAR), play a vital role in preserving genetic diversity. These resources are crucial for the development of climate-resilient varieties and the incorporation of novel traits from wild relatives (Thirumalmurugan et al., 2020).

## 4 Breeding objectives

### 4.1 Yield improvement

**Figure 1:**
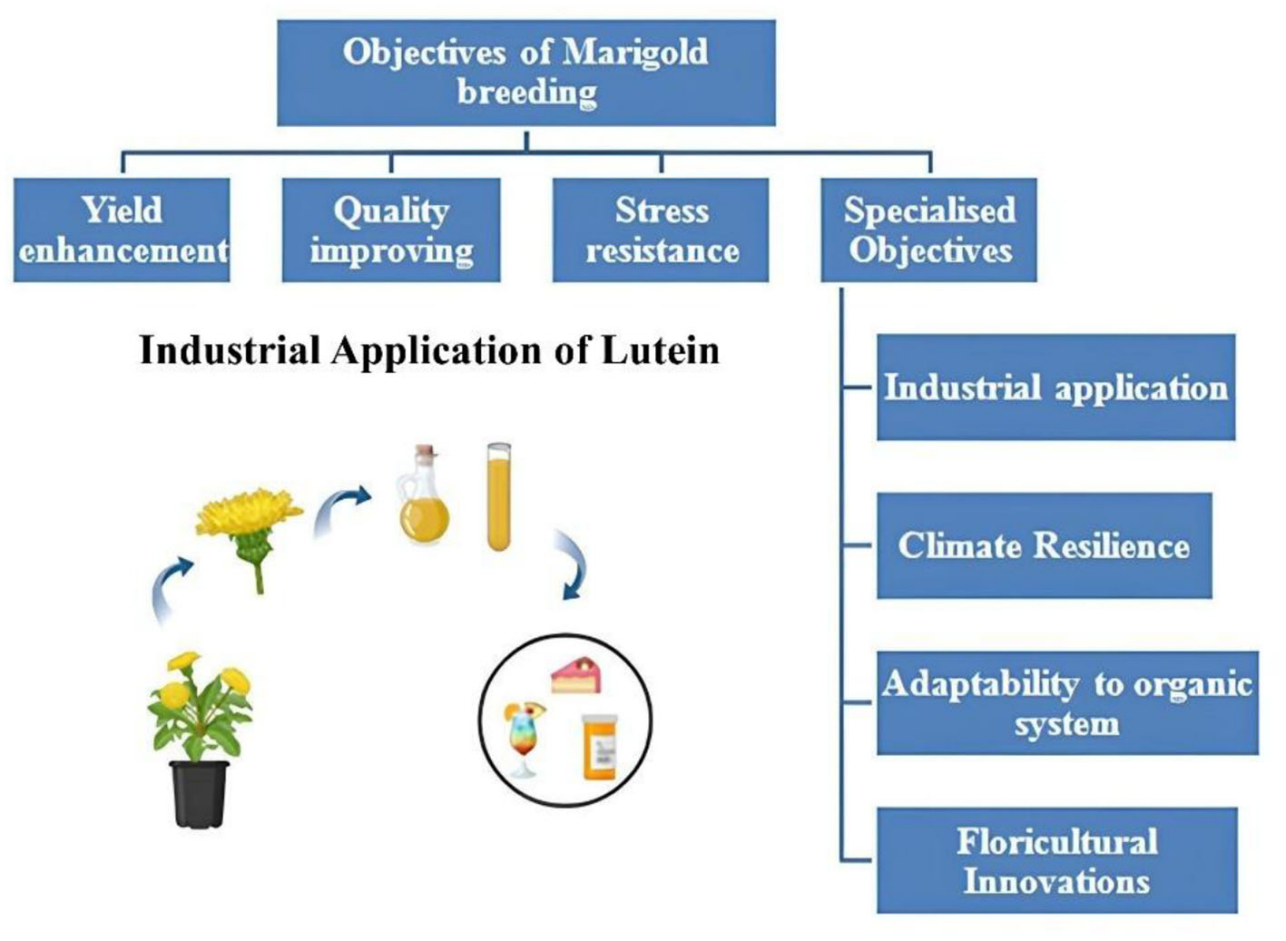
Objectives of marigold breeding.

Yield improvement remains a top priority in marigold breeding due to its direct impact on profitability and market viability. High-yielding varieties are essential to meet the increasing demand for marigold in religious, ornamental, and industrial sectors. Recent research has introduced hybrids such as ‘Arka Abhi,’ ‘Arka Bhanu,’ and ‘Arka Vibha,’ which exhibit significantly improved flower yields, extended shelf life, and better transportability (Thirumalmurugan et al., 2020). These hybrids have shown potential for commercial cultivation in various agro-climatic zones, enhancing farmer incomes and market availability. Breeding programs have also focused on traits such as number of flowers per plant, flower diameter, and plant biomass, which contribute to overall yield. Studies have identified high heritability estimates for these traits, suggesting a strong genetic basis that facilitates effective selection (Adhikary et al., 2025). Marker-assisted selection (MAS) has emerged as a key tool in identifying and selecting genotypes with superior yield potential, enabling the development of hybrids with improved productivity under both irrigated and rainfed conditions (Singh et al., 2022; Joshi et al., 2024). The plant architecture, including growth habits and robust branching, has been optimized in new varieties to maximize flower production (Sumalatha et al., 2023). The overall objectives of marigold breeding are shown in **Figure 1**.

### 4.2 Quality improvement

Flower quality is a multifaceted objective in marigold breeding, encompassing attributes like size, shape, color, fragrance, and shelf life. Varieties with vibrant colors and uniform petal arrangements are highly desired in the ornamental and religious sectors. For instance, *Arka Shubha*, known for its high carotenoid content, has gained popularity for industrial uses such as pigment extraction for food and cosmetics (Sood et al., 2023). Marigolds (*Tagetes erecta* and *Tagetes patula*) are recognized for their medicinal and nutritional value, particularly as a rich source of lutein, a carotenoid beneficial for eye health (González-Morales et al., 2021). Recent advances in genetic transformation have further enhanced breeding efforts, improving germination rates and overall plant quality (Dedhia, 2024). Recent advances in breeding for quality traits have been supported by molecular studies identifying genes like PSY (Phytoene Synthase), PDS (Phytoene Desaturase), ZDS (ζ-Carotene Desaturase), LCY-B (Lycopene β-Cyclase), LCY-E (Lycopene ϵ-Cyclase), CCD (Carotenoid Cleavage Dioxygenase) involved in pigmentation pathways (Cooper and Messina, 2023; Yuan et al., 2023). Studies on the carotenoid biosynthesis pathway has led to the identification of key regulatory genes that enhance color intensity and stability (Toledo-Ortiz et al., 2010). The incorporation of such genes into breeding programs has resulted in varieties with deeper and more vibrant hues (Barbaś et al., 2025). Carotenoids are essential pigments responsible for the red, orange, and yellow colors in many plants (Zhang et al., 2020). The biosynthesis of carotenoids involves several key enzymes, including phytoene synthase (PSY), which catalyzes the first committed step in the pathway, converting geranylgeranyl pyrophosphate (GGPP) into phytoene (Yuan et al., 2015). Subsequent desaturation and isomerization steps are facilitated by enzymes such as phytoene desaturase (PDS) and ζ-carotene desaturase (ZDS), leading to the production of lycopene, a precursor for various carotenoids. The expression levels of these enzymes directly influence carotenoid accumulation and the pigmentation intensity in plant tissues (Toledo-Ortiz et al., 2010). Integrating these regulatory genes into breeding programs has been a successful strategy for developing plant varieties with enhanced pigmentation (Ingkasupart et al., 2015). The overexpression of the PSY gene in transgenic plants has resulted in increased carotenoid content, leading to fruits and flowers with intensified coloration (Yuan et al., 2015). Carotenoids are significant dietary antioxidants and precursors of vitamin A, playing a crucial role in human health by supporting vision, immune function, and overall cellular protection (Sultana et al., 2025). The use of gene editing tools can be made possible by an understanding of the molecular mechanisms supporting the production and control of carotenoid pigments., such as CRISPR/Cas9, to precisely manipulate these pathways (Zhu, 2022). This precision breeding approach enables the development of new varieties with desired pigmentation traits more efficiently and sustainably, meeting both consumer preferences and nutritional needs (Ahmar et al., 2020).

### 4.3 Stress resistance

Stress resistance is a crucial breeding objective for marigold. Biotic stresses, including pest infestations by aphids and thrips, as well as diseases such as Alternaria blight and powdery mildew, can cause significant yield losses. Commercial marigold varieties now have better tolerance to these risks due to breeding operations that have successfully included resistance genes from wild cultivars (Babaei et al., 2021; Oyebamiji et al., 2024). Abiotic stresses, such as drought, salinity, and heavy metal contamination, also adversely affect marigold growth and essential oil quality. Elevated levels of these stresses can result in the production of reactive oxygen species (ROS), which are harmful to plant cells (Riaz et al., 2013). To counteract this, wild marigold utilizes various tolerance mechanisms, such as boosting antioxidant activity to sustain cellular redox balance, increasing lipid peroxidation to protect cell wall integrity, synthesizing secondary metabolites, and accumulating osmolytes. Understanding these physiological and biochemical responses is vital for developing stress-tolerant marigold lines (Kaushal et al., 2024). By incorporating these resistance genes into breeding programs, marigold cultivars that are more tolerant to biotic and abiotic stress have been developed for guaranteed quality and sustainable production. High temperatures, salt, and drought are examples of abiotic stressors that are equally harmful to plant growth and productivity (Guo et al., 2022). Drought stress, affects water use efficiency, photosynthetic activity, and nutrient uptake, leading to reduced flower yield and quality (Ma et al., 2020). Studies have identified drought-responsive genes that regulate key physiological and biochemical pathways, enabling the development of drought-tolerant hybrids (Kumar T. et al., 2023). Similarly, salinity-tolerant varieties are being developed by targeting traits like osmotic adjustment and ion homeostasis, ensuring stable production in saline soils (Ma et al., 2020). Emerging technologies like CRISPR-Cas9 and transcriptomics are further aiding stress-resilience breeding by enabling precise gene editing and detailed analysis of stress-responsive pathways. These tools have facilitated the identification of genes responsible for abiotic stress tolerance, paving the way for the rapid development of resilient varieties (Xu et al., 2020). CRISPR-Cas9, in particular, has revolutionized the targeted modification of genes associated with drought, salinity, and temperature stress tolerance, while transcriptomics provides a comprehensive view of the gene expression changes under various stress conditions (Xu et al., 2020; Verma et al., 2023).

### 4.4 Specialized objectives

Breeding programs are targeting high carotenoid content for pigment extraction and medicinal properties. Varieties with enhanced lutein and zeaxanthin content are being developed to meet industrial demands (Buchori et al., 2024). As climate change introduces new challenges, breeding programs focus on developing varieties adapted to unpredictable weather conditions, such as unseasonal rainfall and extended dry periods (Cai et al., 2024). The demand for organic flowers has spurred breeding programs to develop varieties that thrive under organic cultivation with minimal chemical inputs (Zhang et al., 2022). Breeding for unique traits like double flowers, novel petal shapes, and bi-colored varieties is gaining traction to cater to high-value markets (Zhang et al., 2022).

## 5 Conventional breeding in marigold

### 5.1 Mass selection

Mass selection is a traditional and straightforward breeding method used in marigold cultivation to enhance desirable traits such as flower size, color, and yield. In this approach, a large number of plants exhibiting these traits are selected, and their seeds are bulked together for the next generation (Barut et al., 2023). This method has been effective in developing open-pollinated varieties like ‘Calcutta Orange’ and ‘Calcutta Yellow,’ which are widely grown across India (Adhikari et al., 2020). The Indian Institute of Horticultural Research (IIHR) has developed marigold varieties using mass selection. One notable variety is ‘Arka Pari,’ a French marigold characterized by a dwarf growth habit and high flower production (Dulf et al., 2013). The flowers exhibit an orange hue, transitioning into various shades of orange depending on factors such as light, temperature, and the developmental stage. Blooming occurs year-round, beginning 30 days after planting and lasting for approximately nine weeks (Lenawaty et al., 2022). Furthermore, a study on the genetic diversity of African marigold genotypes assessed 33 genotypes based on 27 traits influencing yield, carotenoid levels, and lutein content. The study found that high heritability coupled with high genetic gain was observed for traits such as the number of secondary branches, fresh petal yield per flower, dry petal yield per flower, dry weight per flower, flower weight per plant, flower number per plant, zea-xanthin content, lutein content, total carotenoid content, seed number per flower, 100 seed weight, number of seeds per gram, and shelf life. This indicates that these traits are likely governed by additive gene effects, making selection effective for their improvement (Kumar A. et al., 2023). While mass selection is effective, modern breeding techniques such as marker-assisted selection (MAS) and *in vitro* regeneration are also being employed to enhance marigold breeding programs. For example, a study validated a SCAR marker linked to genic male sterility in marigold, facilitating MAS breeding programs (Asha et al., 2019). Furthermore, a protocol for *in vitro* regeneration and rapid mass multiplication of apetalous male sterile lines of marigold has been developed, enabling rapid propagation of desirable genotypes (Partap et al., 2023). Marigold varieties released through different breeding methods are listed in **Table 2**.

**Table 2:** Marigold varieties released through different breeding methods.

Sl no	Variety	Species	Released by	Method	Yield
1	Pusa Arpita	French marigold	IARI	It is a selection from heterozygous population of French marigold.	18–20 tons/ha
2	Pusa Bahar	African marigold	IARI	It is an open pollinated variety of African marigold	25–30 ton/ha
3	Pusa Deep	French marigold	IARI	It is an early flowering variety of French marigold	18–20 ton/ha
4	Pusa Narangi Gainda	African marigold	IARI	Open pollinated variety	80 quintals per hectare
	Pusa Basanti Gainda	African marigold	IARI	–	10.735 t ha
5	Arka Bangara	African marigold	IIHR	It is a selection from a germplasm type	18 tons/acre
6	Arka Pari	French marigold	IIHR	–	4.7tons/acre
7	Arka Bangara-2	African marigold	IIHR	–	
8	Arka Agni	African marigold	IIHR	Developed from hybridization	7-7.5 tons/acre
9	Arka Alankara	African marigold	IIHR	–	6-6.5 tons/acre
10	Arka Madhu	French marigold	IIHR	–	5.8 tons/acre
11	Arka Shubha	African marigold	IIHR	–	High carotene content of around 2.8% (for all marigolds, it’s maximum up to 1.4%)
12	Hisar Jafri-1 (Hisar Beauty)	French marigold	Chaudhary Charan Singh Haryana Agricultural University, Hisaar, Haryana	–	It is a dwarf variety suitable for exhibition, bedding and potting
13	Hisar Jafri-2	French marigold	Chaudhary Charan Singh Haryana Agricultural University, Hisaar, Haryana	–	It is suitable for loose flower production besides landscaping.
14	MDU-1	African marigold	TNAU	It is a selection from a germplasm type	41.54 t/ha
15	Punjab Gainda No. 1	African marigold	Punjab Agricultural University, Ludhiana,	–	11 t/ha
16	Bidhan Basanthi (BM-1)	African marigold	BCKV, Kalyani, West Bengal,	Hybridization	8.5-9.0t/ha
17	Bidhan Gold (BM 2)	African marigold	BCKV, Kalyani, West Bengal,	Molecular Breeding	9.5-10t/ha

### 5.2 Pure-line selection

It is a crucial plant breeding method aimed at isolating and propagating genetically superior homozygous lines from a heterogeneous population. In marigold (*Tagetes* spp.), this technique is widely utilized to improve traits such as flower yield, carotenoid content, and plant architecture. Recent research highlights the significance of evaluating pre-breeding lines for desirable attributes (Ghosh et al., 2023; Ayiecho and Nyabundi, 2025). A study by (Singh et al., 2023) assessed various marigold lines for total carotenoid content and flower yield, identifying six pre-breeding lines with high carotenoid accumulation, which are valuable for hybrid development (da Silva and Viana, 2024; Maratovna et al., 2025). Selection techniques like Best Linear Unbiased Prediction (BLUP) and General Combining Ability (GCA) have been evaluated for their effectiveness in parental selection, with BLUP emerging as a dependable method for enhancing breeding success (McGowan et al., 2021; Anilkumar et al., 2022; Liu et al., 2024). Advancements in haploid induction methods, such as doubled haploid production through anther culture (Sharma et al., 2024), have also been investigated as a strategy to facilitate pure line development (Majumder et al., 2018b). Clonal fidelity assessment using microsatellite markers has further validated the uniformity and stability of these doubled haploid lines, reinforcing their role in ensuring genetic purity (Wang et al., 2020). In order to improve the effectiveness and precision of pure line selection in marigold breeding, these results highlight the significance of combining traditional selection techniques with molecular technologies (Majumder et al., 2018b; Biswas and Kumar, 2023; Sumalatha et al., 2023).

### 5.3 Pedigree selection

Pedigree selection is a breeding technique that selects superior individuals over several generations while keeping thorough records of their history in order to improve particular qualities in plants (Alemu et al., 2024; Hansen et al., 2025). In marigold (*Tagetes* spp.), this method is particularly useful for enhancing flower yield, color intensity, disease resistance, and growth habits (Merrick et al., 2022). The process involves selecting the best-performing plants from segregating populations and continuously evaluating their progeny over successive generations (Prasad et al., 2025). Recent studies have highlighted the efficiency of pedigree selection in marigold breeding (De, 2017; Begna, 2021). Pedigree selection has been successfully utilized to improve floral characteristics and resistance to Fusarium wilt, a major disease affecting marigold cultivation (Oyebamiji et al., 2024). It is found that focusing on high-heritability traits, such as flower size and petal count, led to substantial genetic improvements in later generations (Mekapogu et al., 2023). Molecular tools, such as SSR markers, have also been integrated into pedigree selection programs to ensure genetic purity and accelerate the breeding process (Dida, 2022). The success of pedigree selection in marigold depends on several factors, including the heritability of desired traits, the accuracy of selection, and environmental influences (Kumar T. et al., 2023). By maintaining detailed lineage records and continuously evaluating progeny, breeders can develop superior marigold varieties with enhanced ornamental and agronomic traits (Sinha et al., 2023). The integration of molecular markers further enhances selection efficiency, making pedigree selection a valuable strategy for marigold improvement (Ahmad and Anjum, 2018). Advancements in phenotyping tools have significantly enhanced the precision of selection methods in marigold (*Tagetes erecta*) breeding (Hansen et al., 2025). Automated imaging systems now enable breeders to quantify traits such as flower size, petal arrangement, and disease symptoms, thereby reducing subjectivity and increasing efficiency in trait selection (Gao et al., 2021; Jiang et al., 2024). For instance, hyper spectral imaging has been utilized to predict leaf nitrogen content and carbon-to-nitrogen ratios with high accuracy (Gill et al., 2022), underscoring the utility of automated imaging in assessing complex physiological traits in marigold (Ting et al., 2023).

### 5.4 Hybridization

Hybridization involves crossing two genetically distinct parent lines to produce offspring (F1 hybrids) that combine the best traits of both parents. Hybrid vigor, or heterosis, is commonly observed in marigold, resulting in higher yields and improved adaptability. The marigold hybrids ‘Arka Abhi,’ ‘Arka Bhanu,’ and ‘Arka Vibha’ have been developed by the ICAR-Indian Institute of Horticultural Research (IIHR) to enhance commercial cultivation (Sankar and Bharathi, 2021). These hybrids are noted for their high yield, larger flowers, and extended shelf life, making them particularly suitable for garland making, landscaping, and industrial applications. For instance, ‘Arka Vibha’ offers a shelf life of 8–9 days, which is advantageous for prolonged use in decorative purposes (Sankar and Bharathi, 2021). Hybrid seed production involves controlled pollination, including emasculation (removal of male parts) and hand pollination to prevent contamination. Male sterility systems have also been explored to simplify hybrid seed production, although they are not yet widely utilized in marigold breeding. Recent hybrids have shown resistance to pests and diseases, such as powdery mildew and nematodes, making them more sustainable for cultivation. Efforts to combine traits like drought tolerance and high carotenoid content have resulted in hybrids that are both resilient and commercially valuable.

### 5.5 Mutation breeding

Mutation breeding has traditionally been considered a secondary technique, it has gained prominence in recent years for creating novel genetic variability. This method uses mutagens like gamma rays and EMS (ethyl methanesulfonate) to induce changes in the DNA, leading to unique and desirable traits. Induced mutations have been used to develop marigold varieties with rare flower colors, including shades of pink and purple, expanding the ornamental appeal of marigold (Mir et al., 2023). Mutation breeding offers great potential but is a time-consuming process. The identification of useful mutations requires thorough screening and careful selection, which may involve backcrossing or repeated selection cycles.

### 5.6 Challenges in conventional breeding

Conventional breeding methods, though effective, face several challenges including the narrow genetic base of marigold populations in India limits the scope of selection and hybridization. Efforts to introduce genetic material from wild species like *Tagetes minuta* and *Tagetes tenuifolia* are underway but face technical challenges (Huang et al., 2024). Breeding cycles in marigold, particularly for developing hybrids or selecting pure lines, require several years to stabilize desirable traits. Conventional breeding struggles to match the rapid evolution of pests and pathogens, requiring complementary approaches such as molecular breeding.

## 6 Modern breeding approaches in marigold

### 6.1 Marker-assisted selection

Marker-Assisted Selection (MAS) is a powerful tool that leverages molecular markers to identify specific genes or regions of the genome associated with traits of interest. Breeders can efficiently select superior genotypes without relying on lengthy phenotypic evaluations by associating markers with traits like disease resistance, flower quality, and yield (Majumder et al., 2018a). Molecular markers have been used to identify Quantitative Trait Loci (QTLs) linked to key agronomic and ornamental traits in marigold, including flower size, color, and yield. This allows breeders to implement Marker-Assisted Selection (MAS) in early generations, expediting the development of high-quality varieties. For instance, the identification of a Sequence Characterized Amplified Region (SCAR) marker linked to genic male sterility in marigold has facilitated the efficient selection of parental lines in hybrid breeding program (Asha et al., 2019). In marigold, research has aimed at identifying QTLs associated with resistance to biotic stresses, including powdery mildew and nematodes, which pose significant challenges to cultivation in India. The combination of these tools in marigold breeding programs is accelerating the development of varieties with superior traits while reducing the trial and error associated with traditional breeding methods (Asha et al., 2019).

### 6.2 Genomic selection

Genomic selection (GS) is an emerging tool in plant breeding that uses genomic data to predict the breeding value of individuals before they are evaluated for traits (Wang et al., 2020). This approach holds great promise for marigold, particularly in predicting traits like flower yield, carotenoid content, and abiotic stress tolerance (Lahkar et al., 2024). The integration of genomic data, such as single-nucleotide polymorphisms (SNPs), provides breeders with insights into the genetic makeup of marigold lines, enabling them to select individuals with desirable genetic profiles (Barbaś et al., 2025). Genomic selection can significantly reduce the time required to assess breeding lines by predicting their performance based on DNA markers rather than relying solely on phenotypic assessments (Tarakeshwari and Pavan, 2023; Majumder et al., 2018b). While genomic selection has been successfully applied to crops like rice and maize, its application in marigold (*Tagetes erecta*) breeding is still in the early stages (Sudhakaran et al., 2024). Ongoing research aims to identify effective markers for traits of interest in marigold and to develop strategies for incorporating genomic selection into existing breeding programs. In India, where marigold is a significant commercial crop, establishing a genomic selection framework could accelerate breeding progress, particularly in enhancing climate resilience and disease resistance (Mir et al., 2019). The key Genes and GM Technologies Used for Improvement of Flower Color in Marigold are tabulated in **Table 3**.

**Table 3:** Key genes and GM technologies used for improvement of flower color in marigold (*Tagetes erecta*).

No.	Target gene(s)	Function/trait	GM technology used	Results/outcome	Reference
1	CDS (chrysanthemyl diphosphate synthase; from Chrysanthemum cinerariaefolium)	Boost pyrethrin (natural insecticide) biosynthesis in T. erecta	Stable Agrobacterium-mediated transformation of T. erecta hypocotyl explants; CaMV35S promoter; hygromycin selection	Transgenic marigold plants accumulated ~26-fold higher pyrethrins vs. controls (HPLC).	(Godoy-Hernández et al., 2006; Gupta and Ur Rahman, 2015)
2	GFP reporter + bar (phosphinothricin resistance) in binary vector 301vacGFPNM	Demonstrate transgene expression & selectable herbicide resistance; establish practical in-planta method	Agrobacterium-mediated in-planta “seed priming + vacuum infiltration” (no tissue culture); confirmation by PCR, GFP fluorescence, and Basta resistance	Stable T_0_ and T_1_ transformants obtained; optimal conditions reported (e.g., OD600≈1.3, 10-min vacuum); method reduces complexity/time for marigold transformation.	(Godoy-Hernández et al., 2006)
3	GUS (uidA) reporter; hpt selectable marker (vector pIG121-Hm)	Proof-of-concept transformation in male-sterile marigold line	Stable Agrobacterium-mediated transformation of leaf explants	Generated independent transgenic lines expressing GUS in a male-sterile genotype—first report for that single genotype.	(Narushima et al., 2017)
4	GUS (gusA intron) reporter; nptII selectable marker (vector pBI121; strain LBA4404)	Establish efficient regeneration + transformation platform for T. erecta	Stable Agrobacterium-mediated transformation with direct regeneration	Efficient protocol established; stable GUS expression and nptII PCR confirmation in regenerated plants (foundation for trait genes).	(Narushima et al., 2017)
5	GUS (pCAMBIA2301)	Rapid assay for marigold transformability	Agrobacterium-mediated transient transformation (multiple explants)	Transient GUS expression detected within 3 days—useful for vector/construct testing before stable work.	(Gupta and Ur Rahman, 2015)

### 6.3 CRISPR-Cas technology

The CRISPR-Cas9 system, a groundbreaking genome editing tool, has transformed plant breeding by enabling precise and targeted modifications of the plant genome. This approach allows for the incorporation of desirable traits without relying on traditional crossbreeding methods. The CRISPR-Cas technology presents significant potential for addressing challenges such as pest resistance, flower quality, and stress tolerance. Successful applications of CRISPR-Cas9 in ornamental plants, including modifications to flower color in *Petunia hybrida*, demonstrate its versatility (Nishihara et al., 2024). Recent studies have highlighted CRISPR-Cas9ability to enhance consumer-preferred traits in various crops (Verma et al., 2023), indicating that similar strategies could be explored in marigold to modify anthocyanin biosynthesis pathways and develop novel flower colors. Enhancing stress tolerance in marigold is another promising avenue for CRISPR-Cas9 technology. Introducing genes responsible for resistance to abiotic stresses such as drought and salinity could significantly improve the adaptability to harsh growing conditions. Research on other crops has demonstrated that CRISPR-mediated gene editing can strengthen responses to abiotic stress (Yadav et al., 2023), suggesting that similar methods could be applied to marigold for genetic improvement. Silencing genes linked to undesirable traits, such as short flower shelf life or reduced vase longevity, is another potential application of CRISPR. Targeting ethylene biosynthesis or signaling pathways, which are crucial to flower senescence, has been shown to extend vase life in ornamental plants (Xu et al., 2020). Implementing these techniques in marigold could enhance its commercial appeal by increasing flower longevity. Regulatory challenges currently limit the widespread adoption of CRISPR-Cas in India, but as the technology becomes more accessible and widely accepted, its potential for precision breeding in marigold will increase. Future research will aim to refine CRISPR-Cas9 protocols, minimize off-target effects, and navigate regulatory and ethical considerations (Dhanavel, 2022). The Potential Candidate Genes for CRISPR/Cas-Mediated Genome Editing in Marigold is tabulated in **Table 4**.

**Table 4:** Potential candidate genes for CRISPR/Cas-mediated genome editing in marigold.

No.	Gene	Function/trait	Findings	Reference
1	PDS (phytoene desaturase)	Visible albino/bleaching phenotype — routine proof-of-concept target for CRISPR in many plants	Widely used as reporter target in dicots/ornamentals; no marigold CRISPR report yet but applicable.	(Sirohi et al., 2022)
2	PSY, PDS, ZDS, LCY (carotenoid biosynthesis genes)	Modify carotenoid accumulation (lutein/zeaxanthin) — change flower color or lutein content	Marigold carotenoid profiles and expression characterized; good candidate genes for increasing lutein via editing or promoter engineering.	(Zhang et al., 2020)
3	CCD (carotenoid cleavage dioxygenases)	Alter carotenoid cleavage → affect pigment and aroma compounds, lutein degradation	CCD family members identified in marigold transcriptome; manipulation can change flower pigment and aroma profile.	(Liu et al., 2024)
4	MADS-box genes (e.g., AG, AP, SEP family members)	Control floral organ identity, flowering time, and morphology — edit for novel flower forms/colors	Systematic identification of MADS-box genes in marigold genome; potential for editing floral traits.	(Farinati et al., 2023; Liu et al., 2024)
5	Genes controlling male sterility/fertility (e.g., CMS-related genes, meiosis regulators)	Create male sterile lines for hybrid seed production or facilitate breeding	CRISPR widely used to induce male sterility; marigold genome resources enable targeting, though no reports yet.	(Jin et al., 2023)

## 7 Tissue culture and micropropagation in marigold breeding

### 7.1 *In vitro* techniques for rapid multiplication

**Figure 2:**
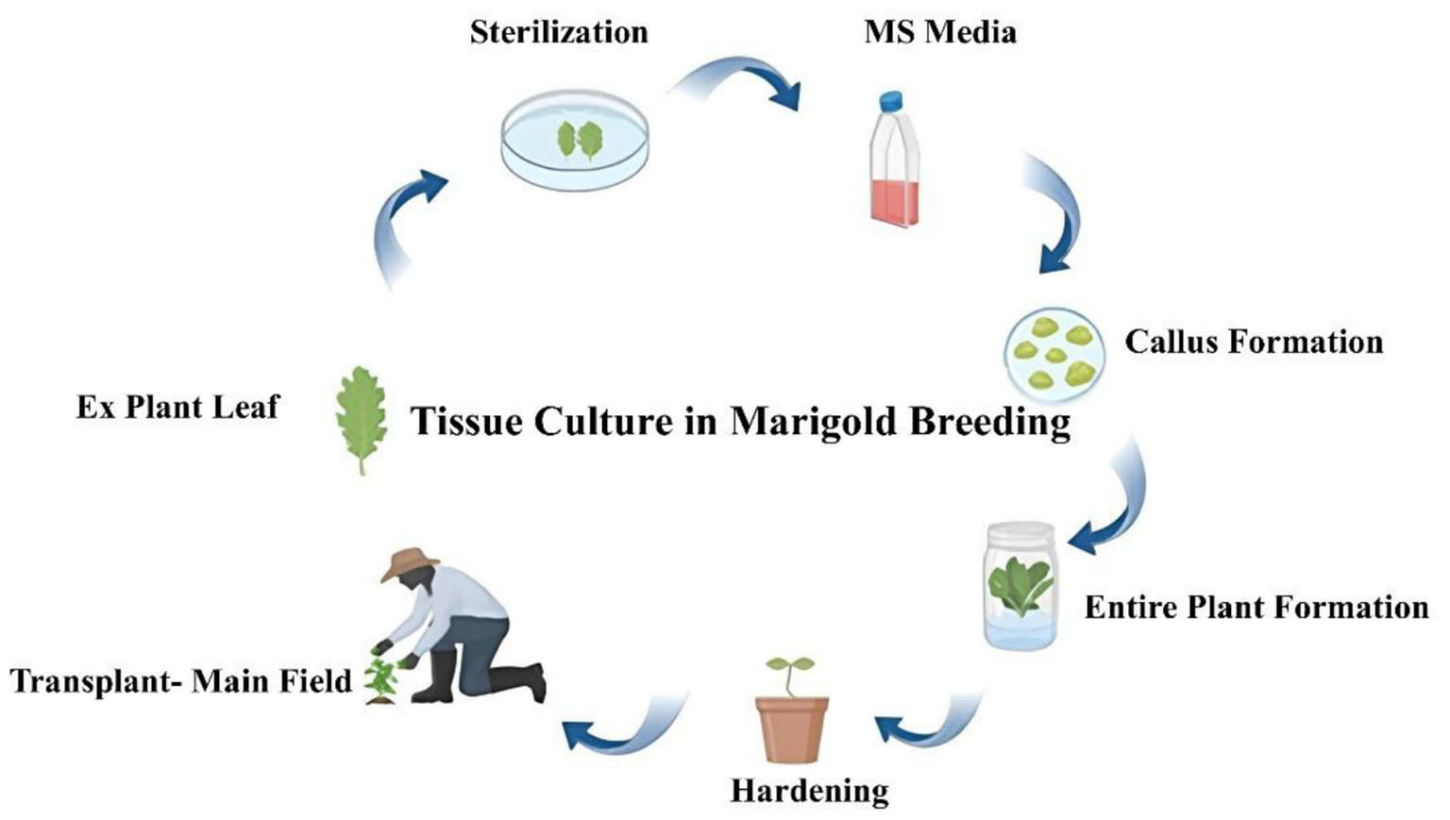
Tissue culture in marigold breeding.

Tissue culture, specifically *in vitro* propagation, offers an efficient method for mass production of marigold plants. It bypasses the traditional seed propagation method, ensuring that desirable traits are consistently reproduced across generations. Recent advancements have allowed for the establishment of protocols for the regeneration of marigold from different explants such as shoot tips, leaf pieces, and flower tissues. Tissue culture and micropropagation techniques have important tools in modern marigold breeding programs. These methods offer rapid and efficient means for propagating elite genotypes, preserving genetic diversity, and enhancing breeding strategies (Kumar et al., 2018). Micropropagation has been employed extensively for the clonally multiplication of elite marigold genotypes with desired characteristics. This method allows breeders to obtain genetically uniform plants quickly and in large numbers. A key advantage of *in vitro* propagation is its ability to overcome the limitations of seed propagation, such as seed dormancy and low germination rates, which are sometimes seen in marigold cultivars. For instance, the use of cytokinin-based media has been shown to enhance shoot multiplication in marigold cultivars, leading to a rapid increase in plant numbers (Fibriani et al., 2023). The outline of tissue culture in marigold is shown in **Figure 2**.

### 7.2 Somaclonal variation in marigold

Somaclonal variation refers to the genetic variation observed in regenerated plants arising from changes in genetic material during *in vitro* culture. These variations can appear as changes in phenotypic traits, such as flower color, size, or disease resistance (Chen and Henny, 2003). The potential of somaclonal variation for developing new marigold cultivars with enhanced traits, such as improved yield, stress tolerance, and disease resistance, has been extensively studied. Somaclonal variation has been effectively utilized to develop drought-tolerant marigold lines (Mehraj et al., 2019). demonstrated that exposing marigold tissue cultures to osmotic stress using mannitol led to the selection of drought-tolerant genotypes. These selected clones exhibited higher proline and soluble sugar content, contributing to enhanced drought tolerance. Such drought-resistant lines are particularly valuable in regions facing water scarcity, ensuring sustainable marigold cultivation (Ferreira M dos, 2023). In addition to drought tolerance, somaclonal variation has also been successfully employed to develop marigold lines with enhanced pigment content, specifically higher levels of carotenoids, which are important for their ornamental and industrial value (Sarkar et al., 2016). It is also found that through somaclonal variation, marigold cultivars with significantly higher carotenoid content were developed, improving both the ornamental appeal and industrial value for uses in pharmaceuticals and natural dyes (Ferreira M dos, 2023). In chrysanthemums, has demonstrated that somaclonal variation can lead to desirable traits, including extended vase life and delayed senescence and a study on *Chrysanthemum × morifolium* protoplast regenerants observed significant variations in flower longevity and delayed senescence among the regenerants (Eeckhaut et al., 2020).

### 7.3 Applications of somaclonal variation in marigold

Somaclonal variation has played a crucial role in enhancing various traits in marigold breeding. In addition to improving stress tolerance and pigment content, it has been instrumental in increasing resistance to biotic stresses, including fungal infections, nematodes, and viral diseases. It is demonstrated that somaclonal variation in marigold resulted in greater resistance to fungal pathogens such as *Fusarium oxysporum* and *Botrytis cinerea*, both of which pose significant challenges to marigold cultivation (Dhanavel, 2021). This technique has also contributed to the economic growth of marigold farming by generating genetic diversity through tissue culture. As a result, breeders have successfully developed cultivars with novel flower colors and shapes, enhancing their market appeal. Reports indicate that somaclonal variation has led to the introduction of new marigold cultivars with distinctive flower traits, such as bi-colored petals, which cater to specialized ornamental markets (De, 2017). Somaclonal variation has also been utilized to enhance essential oil production in marigold. Studies indicate that inducing genetic variation in marigold lines has resulted in significant increases in essential oil yield, adding greater value to the flowers for the perfume and aromatherapy industries.

### 7.4 Challenges and limitations of somaclonal variation

While somaclonal variation presents a valuable opportunity for improving marigold traits, there are challenges associated with its application. One of the primary concerns is the stability of the traits induced through somaclonal variation. In some cases, the traits exhibited by somaclonal variants may not be stable across generations, resulting in a loss of desirable characteristics over time (Kumar, 2017). This poses a challenge for breeders who rely on stable traits for commercial production. Another limitation is the potential for undesirable traits to emerge as a result of somaclonal variation. While beneficial traits such as enhanced drought tolerance or improved pigment content may be induced, unwanted traits, such as reduced flower yield or poor plant architecture, can also emerge in the process (Zhang et al., 2020). Therefore, extensive evaluation and selection are required to ensure that somaclonal variants maintain the desired characteristics and remain stable over multiple generations. It is also emphasized the need for careful screening and long-term evaluation to ensure that somaclonal variants meet the rigorous standards required for commercial marigold production (Cicevan et al., 2022).

## 8 Achievements in marigold breeding

### 8.1 High-yielding varieties

**Figure 3:**
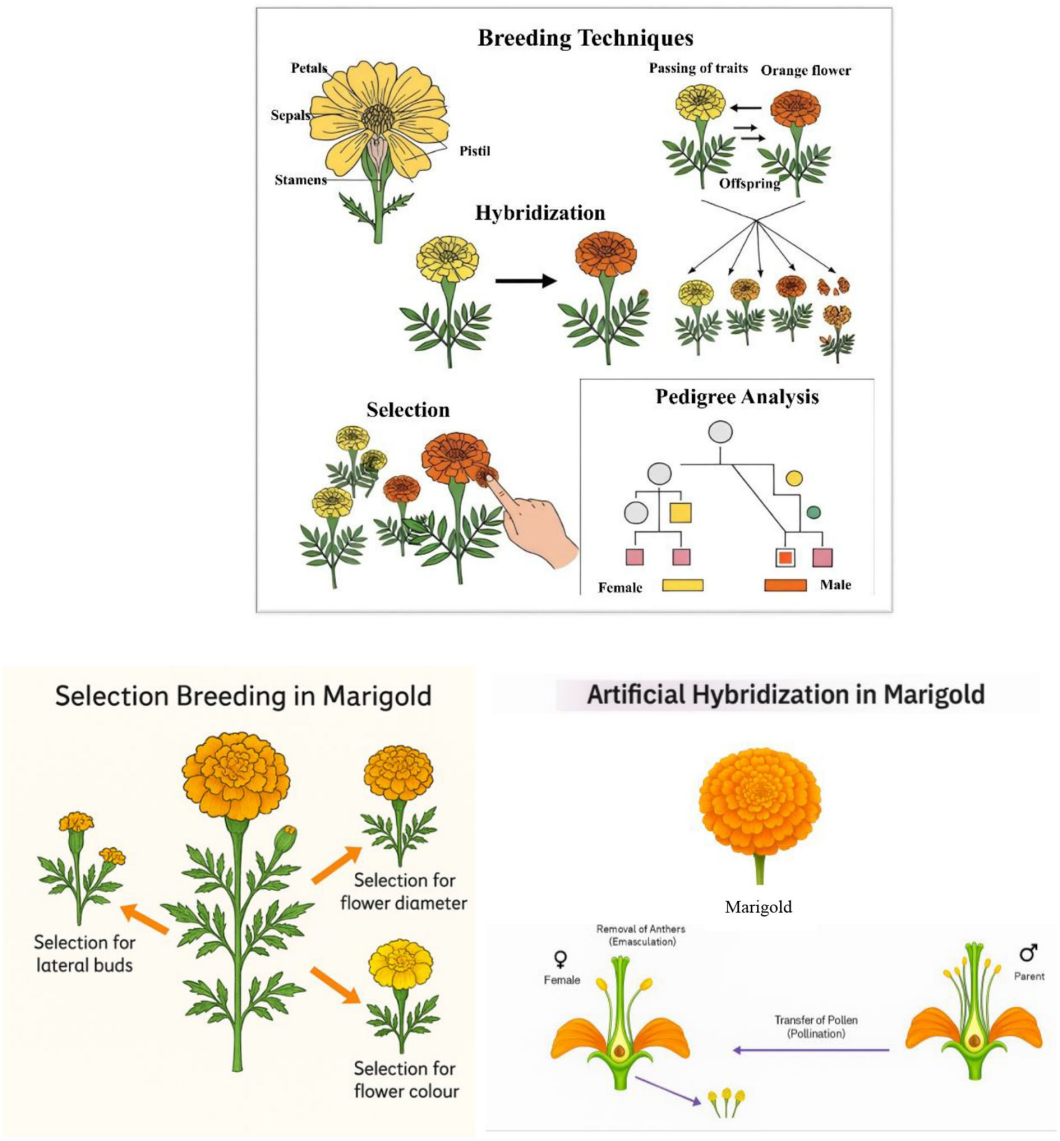
Different conventional breeding techniques followed in Marigold.

Marigold breeding in India has achieved significant milestones over the years, especially in areas such as yield enhancement, quality improvement, and stress tolerance. The integration of both conventional and modern breeding techniques has led to the development of marigold cultivars that meet the increasing demands of the ornamental and pharmaceutical industries (Tang et al., 2020). One of the main goals of Indian breeding initiatives has been to create marigold cultivars with high yields. As the demand for marigold flowers continues to rise, particularly for use in religious ceremonies, decorations, and the extraction of carotenoids for industrial purposes, there is a growing need for varieties that offer high production efficiency and consistent yield (Youssef et al., 2020). One of the notable achievements in this area is the release of hybrid varieties such as ‘Arka Bangara,’ ‘Arka Bangara-2,’ and ‘Arka Agni,’ which have demonstrated significantly higher flower yields compared to traditional cultivars. It is reported that these hybrids have been specifically bred to enhance flower size, flower count per plant, and overall yield per hectare, contributing to greater productivity in marigold cultivation (Kumar et al., 2019; Panda et al., 2022). Furthermore, these varieties are known for their improved disease resistance, which has enhanced their adaptability in various climatic conditions across India. Breeding for high yield involves the selection of plants with desirable traits, such as larger flowers and increased branching (Kumar et al., 2019). Recent advancements in hybridization techniques have enabled the development of marigold cultivars that not only exhibit higher yields but also have enhanced shelf life, making them more commercially viable. It is highlighted the potential of hybrid vigor in marigold, where F1 hybrids display superior growth performance and resistance to biotic stresses (Modi et al., 2009). These hybrids have been widely adopted in marigold production, especially in states like Karnataka, Maharashtra, and Tamil Nadu, where marigold farming is economically significant. Different breeding techniques and Marigold varieties released in India are tabulated in **Table 1** and **Table 5**. Different conventional breeding techniques followed in Marigold is shown in **Figure 3**.

**Table 5:** Breeding techniques and marigold varieties released in India.

Breeding technique	Example varieties released in india
Hybridization	Arka Agni, Arka Bangara-2, Arka Ambara, Arka Orange (IIHR); Maria 91, Rupa, Sakura 031, Royal Orange (evaluated hybrids in India)
Pedigree/Selection	Pusa Narangi Gainda, Pusa Basanti Gainda, Pusa Bahar, Pusa Deep, Pusa Arpita (IARI)
Mutation Breeding	Pusa Centenary (IARI); Mutant selections derived from Pusa Narangi Gainda and others (gamma-ray induced mutants)
Clonal Selection/Vegetative Propagation	Arka Bangara, Arka Bangara-2 (IIHR); Bidhan Marigold BM-1, BM-2, BM-3 (BCKV)
Regional Selection/State Releases	Bidhan Marigold series (BM-1, BM-2, BM-3 — BCKV, West Bengal); Siracole (OUAT/OARI, Odisha); Arka Agni and Arka Bangara-2 (recommended regionally); Coimbatore Local selections (TNAU, Tamil Nadu)
Male-Sterile Lines/Biotech-Based Breeding	Male-sterile breeding lines (IIHR, IARI) used for hybrid seed production; tissue-culture derived lines for multiplication

### 8.2 Quality enhancement

Breeding programs in India have also focused on improving the quality of marigold flowers, especially their aesthetic and biochemical properties. Market demands for high-quality marigold flowers with improved color, size, and shelf life have led to the development of several superior cultivars (Lenawaty et al., 2022). Quality traits such as vibrant color, longer vase life, and higher carotenoid content have been prioritized in recent breeding efforts. Varieties like ‘Arka Shubha,’ known for its high carotenoid content, have been developed with industrial applications in mind. These varieties offer high yields of carotenoids, which are important for use in the pharmaceutical, food, and cosmetic industries (Ördögh, 2021). It is found that ‘Arka Shubha’ produced significantly higher levels of lutein and zeaxanthin, which are valuable carotenoids known for their antioxidant properties. These carotenoids have widespread applications in supplements, skincare products, and natural food colorants (Udchachon et al., 2021). In addition to carotenoid enhancement, flower characteristics such as color, size, and shelf life are crucial for meeting the demands of the cut flower industry. Research has led to the development of marigold cultivars with improved flower colors, ranging from deep orange to bi-colored variants, which cater to different market preferences. Ethylene, a plant hormone, plays a pivotal role in regulating processes such as flower wilting and petal drop. Inhibiting ethylene perception or action has been shown to extend the longevity of cut flowers and reduce post-harvest losses. The use of 1-methylcyclopropene (1-MCP), an ethylene action inhibitor, has successfully extended the vase life of several ornamental species by delaying ethylene-triggered aging (Baron et al., 2021). Recent studies have demonstrated that continuous application of 1-MCP during transport and storage can further enhance the post-harvest quality by blocking ethylene action and reducing botrytis incidence. Combining 1-MCP with antimicrobial agents, such as ajowan essential oil or silver nanoparticles, has been found to synergistically improve vase life and delay senescence in gerbera cut flowers (Baron et al., 2021).

### 8.3 Stress tolerance

Abiotic stress tolerance, particularly drought resistance, has been a major focus of marigold breeding programs due to the increasing unpredictability of weather patterns and water scarcity issues in many marigold-growing regions (Mohammed, 2025). Marigold, being a drought-sensitive plant, faces significant challenges in maintaining growth and flower production under water-limited conditions. However, recent research has led to the development of marigold lines exhibiting improved drought tolerance, a critical achievement in ensuring stable production (Hemmati et al., 2018). Research showed that drought stresses result in physiological and chemical changes in marigold plants, such as decreased production of essential oils and flower yield. In response to these challenges, breeders have worked to develop drought-tolerant varieties through conventional selection and genetic improvement (Riaz et al., 2013). The physiological responses of marigold to drought stress and identified key traits associated with drought tolerance, such as improved water use efficiency, osmotic adjustment, and increased root biomass (Eghlima et al., 2024). By selecting and propagating plants with these traits, they were able to develop marigold cultivars that are more resilient under drought conditions (Srinivasan and Jeyakumar, 2018). These cultivars not only exhibit better growth under limited water availability but also produce higher yields of essential oils, which is a major economic benefit. Moreover, the development of marigold cultivars with enhanced tolerance to salinity has also been an area of focus. It is found that marigold varieties like ‘Arka Agni’ exhibit superior tolerance to salinity stress, maintaining better physiological functioning and flower yield under saline conditions (Moradian et al., 2023). This achievement is important for regions where soil salinity is a limiting factor in agricultural productivity.

## 9 Challenges in marigold breeding

The narrow genetic diversity among cultivated marigold varieties remains a significant obstacle for breeders. Most commercial marigold varieties are derived from a limited pool of germplasm, which restricts the genetic variability available for breeding programs. This lack of diversity restricts the development of new traits, including disease resistance and tolerance to abiotic stresses (Bakshi and Ghosh, 2022). Efforts to incorporate wild germplasminto breeding programs are ongoing to broaden the genetic base. Wild relatives are valuable sources of traits such as pest resistance, higher carotenoid content, and adaptability to harsh environments (Patel et al., 2018). Moreover, preliminary breeding activities, including the development of intermediary populations, are essential for integrating wild germplasm into breeding programs. The production of marigolds is being challenged by climate change, which increases abiotic factors like heat, salinity, and drought. Marigold plants are sensitive to temperature fluctuations, which can affect flower size, color, and yield. Drought stress, in particular, has been shown to reduce essential oil content and overall plant vigor (Sachin and Homraj, 2021). To mitigate these effects, breeders are focusing on developing climate-resilient varieties. The primary challenge lies in simultaneously improving stress tolerance and maintaining high yield and flower quality. Marker-assisted selection (MAS) and genomic selection are being explored to accelerate the development of climate-resilient marigold varieties (Krzymińska et al., 2022). Marigold is susceptible to a range of pests and diseases, including Alternaria blight and aphids, which can cause significant yield losses. The limited genetic base has constrained the development of resistant varieties. Incorporating resistance genes from wild species and utilizing molecular tools like CRISPR-Cas for targeted genome editing are promising strategies (Shafiee et al., 2023).

## 10 Future prospectives

The future of marigold breeding holds exciting prospects, driven by advancements in genomics, biotechnology, and sustainable farming practices. One of the primary focuses will be the integration of genomic tools such as marker-assisted selection (MAS) and CRISPR-based genome editing, allowing breeders to develop marigold varieties with specific traits like enhanced carotenoid content and stress tolerance (Namita et al., 2013). The breeding of climate-resilient varieties will become increasingly important to address challenges like drought, salinity, and extreme temperature fluctuations. These varieties will be designed to thrive under changing environmental conditions, ensuring consistent production in diverse climates. Development of marigolds with pest and disease resistance will be a priority, especially in response to rising threats from pests like aphids and diseases such as powdery mildew (Burlec et al., 2021). Marigold breeding will also adapt to the growing demand for organic and sustainable practices, developing varieties suitable for low-input systems and resistant to organic pest control methods. Carotenoid production is also a key focus, as marigolds serve as a major source of natural pigments used in food, pharmaceuticals, and cosmetics (Aswath and Aswath, 2018). Breeders will focus on developing cultivars with higher carotenoid yields without compromising flower quality or stress resistance. Floricultural innovations will serve the ornamental market by producing marigolds with unique flower shapes, bi-colored petals, and improved post-harvest quality, such as longer shelf life and enhanced vase life. Advances in artificial intelligence (AI) and high-throughput phenotyping will streamline breeding efforts, enabling quicker identification of desirable traits and more precise breeding decisions. In the future, marigold breeding will integrate modern technology and environmentally friendly methods to satisfy the changing demands of the medicinal, agricultural, and ornamental sectors, providing a robust and superior crop for a variety of uses.

## 11 Conclusion

Marigold breeding in India has made significant progress over the years, contributing to the enhancement of yield, flower quality, and resistance to pests and diseases. As one of the most economically and culturally important ornamental crops, marigold holds immense potential for expanding its role in both domestic and international markets. However, challenges such as limited genetic diversity, the effects of climate change, and the need for improved pest and disease resistance continue to impede the full realization of its potential. The limited genetic diversity of cultivated marigold remains a major challenge, restricting opportunities for further enhancement. Modern breeding technologies, including marker-assisted selection (MAS) and genomic selection, provide valuable tools for accelerating genetic improvement in marigold. By identifying quantitative trait loci (QTLs) associated with desirable characteristics, these methods enhance selection efficiency. Genome editing techniques such as CRISPR-Cas offer the ability to introduce precise genetic modifications, strengthening marigold’s resistance to both biotic and abiotic stresses. Tissue culture and micro propagation have also proven beneficial in the rapid multiplication of elite genotypes, with the exploitation of somaclonal variation contributing to the development of marigold variants with enhanced stress tolerance and improved pigmentation. Developing climate-resilient varieties capable of withstanding these environmental challenges is a priority for future breeding programs. In conclusion, while considerable progress has been made in marigold breeding, ongoing research and the integration of modern breeding techniques are crucial to overcoming current challenges. Expanding genetic diversity, enhancing climate resilience, and improving pest and disease resistance, marigold breeding will play a pivotal role in ensuring the long-term sustainability and profitability of marigold cultivation in India.
